# Use of Cemented Hemiarthroplasty for Femoral Neck Fractures

**DOI:** 10.5435/JAAOSGlobal-D-24-00183

**Published:** 2025-01-03

**Authors:** Sheena J. Amin, John K. Krumme, L. Nathan Gause, Jonathan R. Dubin, Akin Cil

**Affiliations:** From the Department of Orthopaedics, University of Missouri-Kansas City, Kansas City, MO (Dr. Amin, Dr. Krumme, Dr. Gause, Dr. Dubin, and Dr. Cil), and the Department of Orthopaedics, Kansas City Orthopaedic Alliance, Leawood, KS (Dr. Krumme).

## Abstract

Geriatric femoral neck fractures are common orthopaedic injuries, which are associated with a high morbidity and mortality. Arthroplasty is the optimum treatment for many of these injuries, but debate exists regarding optimal surgical strategy. Multiple recent investigations have demonstrated strong superiority for cemented stems as compared with noncemented fixation with a decreased risk of periprosthetic fracture, shorter length of stay, lower cost, and decreased rate for revision surgery. The main purpose of this article is to refamiliarize the resident or practicing surgeon with cemented arthroplasty by reviewing the basic science of cement, common cementing concerns, and outcomes, as well as by providing tips on cementing technique to ensure safe, simple, and reproducible results.

Geriatric femoral neck fractures are commonly associated with high morbidity and mortality. The incidence of femoral neck fractures in the United States is more than 300,000 each year.^1^ Arthroplasty is the optimum treatment for these injuries, but debate exists regarding the use of cemented or press-fit stems. Recent investigations have demonstrated strong superiority for cementing stems, finding decreased risk of periprosthetic fracture, shorter length of stay, lower cost, and decreased rate for revision surgery.^2,3^ This led the AAOS to publish a Clinical Practice Guideline upgrading its recommendation from “moderate” to “strong” support for the use of cemented stems for hemiarthroplasty in the setting of a femoral neck fracture.^4^ With the new data and clinical guidelines, there has been a recent uptrend regarding the use of cement during hemiarthroplasty for femoral neck fractures. However, as of 2021, only half of all patients with hip fractures treated in the United States underwent cemented hemiarthroplasty.^5^ Given the preponderance of evidence, noncemented stems continue to be used. Perhaps, reasons could include that cementing can be technically challenging, and many surgeons are likely unfamiliar, or uncomfortable, with the technique due to little exposure during training. There is also the concern that cement can impart deleterious effects to healthcare workers, such as irritation to the respiratory tract and eyes as well as an unknown risk to the fetus during pregnancy. The use of cement can also make revision surgery more challenging. The main purpose of this article is to refamiliarize practicing surgeons with cemented arthroplasty. This review will discuss the basic science of cement, common cementing concerns, and outcomes and provide tips on cementing technique to ensure safe, simple, and reproducible results.

## Basic Science of Cementing

Polymethyl methacrylate (PMMA) was first discovered by Otto Rohm in 1901, ultimately commercializing it as Plexiglas (Plexiglas). Because of its handling characteristics and biocompatibility, PMMA was later introduced for a variety of medical uses, including contact lenses and filling of cranial defects.^6^ In 1958, Sir John Charnley initiated the modern era of hip arthroplasty by successfully employing PMMA to fixate a metal prosthesis into the proximal femur, noting that “the technique is considered justifiable in elderly patients where the medullary canal is large and the cortex of the femur is thin and brittle.”^7^

PMMA bone cement is formed by mixing the white, polymer powder with the clear monomer liquid.^8^ Manufacturers will add copolymers to modify physical characteristics of the cement and typically include a catalyst, such as benzoyl peroxide, and a radio-opacifier, such as barium sulphate.^6^ A small volume of toluidine is added as an initiator to liquid monomer.^6^ The resultant chemical reaction is exothermic, with peak temperatures achieving between 70 and 120 degrees Celsius, in vitro.^9^ Collagen denatures with prolonged exposure above 56 degrees Celsius, which raises concerns regarding thermal damage.^9^ However, in vivo studies suggest that temperatures rarely surpass 48°C for longer than a couple minutes.^9^

Cement transmits load from the implant to bone, acting as an elastic buffer between the rigid implant and the softer bone.^10^ The upper end of Young's modulus of cancellous and cortical bone is 1 and 20 GPa, respectively, whereas a cobalt-chromium implant is nearly 220 GPa.^9^ Cemented implants allow for load to be transmitted over a 65 times greater area than noncemented calcar-bearing designs, perhaps decreasing the risk of fracture.^9^ Cement is also viscoelastic, allowing for material creep to occur with constant loading. A polished stem will, therefore, subside slightly in the cement, protecting the important bone-cement interface, with mean subsidence ranging between 1 and 2 mm over the course of 10 years.^11^

**Figure 1:**
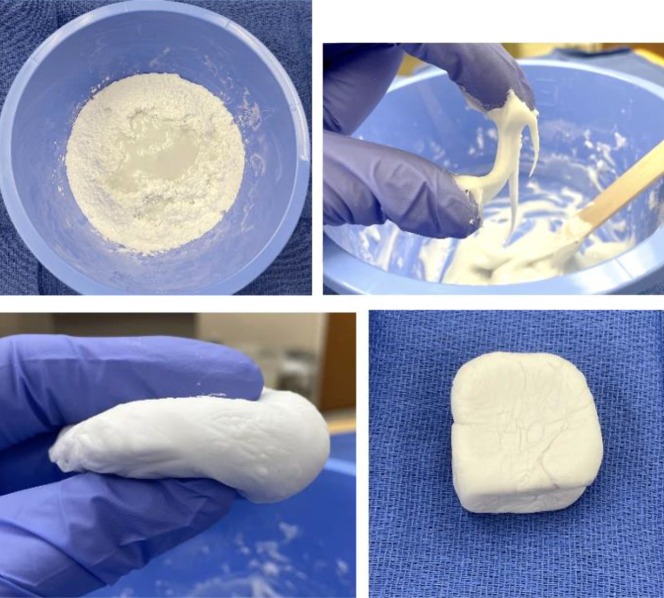
Images showing the process of cement curing involves four stages: (**A**) mixing phase (top left), (**B**) “sticky” or waiting phase (top right), (**C**) working (bottom left), and (**D**) hardening (bottom right), with the entire process taking between 10 and 20 minutes.

The process of cement curing occurs in four stages: (1) mixing, (2) “sticky” or waiting, (3) working, and (4) hardening, with the entire process taking between 10 and 20 minutes, depending on the manufacturer's specific formulation and ambient room temperature (Figure 1).^10,12^ During the mixing phase, typically 60 to 90 seconds, the prepolymerized PMMA, MMA, and catalyst are mixed until there is a homogenous mixture that can be easily stirred. The waiting stage is next, continuing until the cement takes on a duller sheen and no longer sticks to a gloved finger. Cement can be inserted into the femoral canal during the doughy, working phase, which occurs approximately 2 to 4 minutes after the start of the mixing phase.^13^ Ultimately, hardening occurs over the next 1 to 2 minutes, with its duration being dependent on room temperature and air humidity. Warmer ambient temperatures and lower humidity expedite setting time.^10^ “Low viscosity” cements have a longer waiting phase with a short working time, whereas “high viscosity” cements are the opposite. To ease injectability of the cement and increase setting time, surgeons can elect to use a low viscosity cement. The room temperature and humidity can also be controlled to alter setting time.

## Cemented Versus Noncemented Stems

Multiple clinical investigations and national registry analyses have compared the outcomes of cemented and noncemented femoral stems in hemiarthroplasty after a femoral neck fracture. The use of cemented hemiarthroplasty has been consistently associated with less pain, better functional outcomes, lower rates of periprosthetic fracture, and a lower frequency of revision in comparison to noncemented hemiarthroplasties.^3,13-17^ These findings demonstrated generalizability across global populations and the fragility-fracture age range. Fernandez et al^17^ conducted a multicenter, prospective, randomized, controlled trial of patients >60 years of age with femoral neck fractures and found patients receiving cemented stems had higher EuroQol Group-5 Dimension scores at 4 months postoperation. In Australia, Blythe et al^2^ observed that cemented arthroplasty improved outcomes in each age group, <75, 75 to 80, and >85 years of age. Okike et al^18^ conducted a retrospective review of 12,491 patients >60 years of age in the United States who underwent hemiarthroplasty for femoral neck fractures and found that noncemented stems were associated with a markedly higher risk of aseptic revision, 3% versus 1.3% for cemented fixation. Although modern noncemented stems have reduced the risk of intraoperative fracture, there is still a markedly decreased risk of intraoperative fracture with the use of a cemented stem.^1,3^ Furthermore, fracture patterns associated with posterior or posteromedial comminution of the femoral neck could compromise the fixation of a press fit metaphyseal fitting stem or may serve as a stress riser, which may increase the risk of a proximal periprosthetic fracture during or after surgery.

Several well-designed studies do provide contrarian evidence, sparking debate regarding the true benefit of cementing over noncemented. DeAngelis et al conducted a randomized, controlled trial in the United States from 2005 to 2008 to evaluate the outcomes of cemented and noncemented hemiarthroplasty, discovering no difference in functional outcomes between the two groups.^19^ Several investigators have even reported increased mortality, up to 2.3 times, on the day of surgery in patients undergoing the cemented technique due to bone cement implantation syndrome (BCIS), which will be discussed later on.^20^ However, long-term studies constantly unveil no notable difference in mortality between the two techniques.^1,17,18,20-22^

In 2021, to better guide practicing orthopaedic surgeons, the AAOS convened an expert work group that developed an evidence-based clinical practice guideline regarding the management of femoral neck fractures in older adults. They provided the strongest recommendation in support of cementing femoral stems, citing consistent evidence from “2 or more high-quality studies.”^4^ This recommendation has been further endorsed by the American Association of Hip and Knee Surgeons, the Orthopaedic Trauma Association, and other professional organizations.

## Common Cementing Concerns

Given the benefits of cemented hemiarthroplasty, there has been an increasing shift toward the practice in United States. The American Joint Replacement Registry 2022 annual report stated that almost half of all femoral neck fractures treated with hemiarthroplasty were cemented, an increase from the 39% in 2016.^5^ However, despite knowing the benefits of cemented hemiarthroplasty, several concerns have been cited among surgeons who continue to perform press-fit femoral fixation in the setting of fragility femoral neck fracture. These reasons include increased surgical time, BCIS, potential teratogenic or other deleterious effects to health care workers, and challenging revision surgery should it become necessary.^18^

BCIS is described as a transient episode of oxygen desaturation, hypotension, and increased pulmonary arterial resistance leading to cardiovascular collapse. Rutter et al^23^ evaluated the risk of severe harm or death associated with cemented hemiarthroplasty, reporting that out of approximately 180,000 cemented hemiarthroplasties, 21 instances of severe harm (0.01%) and 41 deaths (0.02%) occurred. Of these 62 cases (0.03%), 55 occurred within a few minutes of cementing.^23^ The dire consequences may dissuade some surgeons from using cement; however, the incidence of BCIS is exceedingly rare, and modern-day cementing techniques discussed later in this article may reduce its risk.^8^ Regardless, we recommend informing the anesthesia team at the start of cement application, following the Food and Drug Administration (FDA) recommendation to pay close attention to the patient's hemodynamic status and being ready to administer resuscitative measures in the case of BCIS.

Concern for the safety of healthcare providers is also important. As part of product labeling, the FDA warns against “excessive exposure to the concentrated monomer vapors, which may produce irritation of the respiratory tract, eyes, and possibly the liver. Personnel wearing contact lenses should not be near or involved in mixing this bone cement.”^24^ They also recommend against gloved hands contacting the liquid monomer until the cement is in the doughy phase, as it is a powerful lipid solvent, possibly destroying the glove and damaging underlying tissue.^24^ Of greater concern to women of child-bearing age is its potential teratogenicity. Fetal toxicity is unknown, and neither the FDA nor American College of Obstetrics and Gynecology provide formal guidance. However, in a survey conducted by Harper et al,^25^ 41.7% of female orthopaedic surgeons reported that they would leave the operating room during cementing if they were pregnant and 23.7% would leave if they were breastfeeding. These risks may also dissuade some surgeons from cemented hemiarthroplasty.

Perhaps, an understated impediment to surgeons cementing stems is a lack of familiarity. Ryan et al surveyed orthopaedic surgery residency graduates in 2021 about their perception of training in femoral cementation techniques. Only 42% reported participating in 15 or more cemented hemiarthroplasty procedures during residency. Not surprisingly, the same percentage reported that they intend to cement most of the hemiarthroplasty stems they implant in practice.^26^ This questions whether orthopaedic surgery residents obtain sufficient training on cementing techniques, decreasing their comfort when out in practice.

## Cementing Techniques

Best practices in cementing have improved since Sir John Charnley first described his method in the 1960s. First-generation cementing techniques involved manual mixing followed by digital insertion of cement into an essentially unprepared femoral canal. Second-generation techniques progressed to include prepping the canal with a pulsatile lavage, followed by packing and drying of the canal.^8^ This allows for removal of debris and facilitates interdigitation of the cement at the bone interface. A cement restrictor was then inserted into the canal about 1.5 cm distal to stem tip creating a compartment. A cement gun was also incorporated to insert cement in a retrograde fashion.

Third-generation cementing techniques subsequently used epinephrine-soaked sponges, vacuum mixing, and pressurization of the cement (Video 1).^10,12,13^ Epinephrine-soaked sponges remove the remaining blood, if there are no medical contraindications, decreasing the risk of lamination. Vacuum mixing and pressurization of cement decreases the porosity and improves penetration of the cement into bone.

## Our Institutional Experience With Cementing

**Figure 2:**
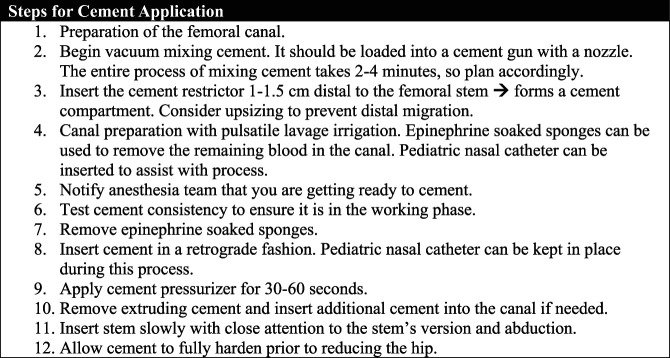
Flowchart showing the steps of cement application with recommended tips used by the arthroplasty surgeons at our institution.

Despite the AAOS clinical guidelines, some surgeons at our institution prefer press-fit stems for hemiarthroplasty because of several concerns cited in this article. We will discuss the cementing technique used by our lead author who is a fellowship-trained arthroplasty surgeon (Figure 2).

To start, we recommend instituting a dedicated training for the surgical technicians on cementing techniques. Trying to use these specialized personnel for cases ameliorates some of the stress associated with cementing for the surgeon, although it is certainly not mandatory nor always feasible. We prefer to use a low-viscosity cement, plus or minus prophylactic antibiotics. For stems, typically our lead author prefers a taper wedge polished stem, which allows for controlled subsidence and force distribution. However, composite beam stems that have a collar and are grit blasted to avoid subsidence are also acceptable. Stems with canal centralizers distally improves appropriate placement.

After making the appropriate femoral neck cut, we typically follow modern third-generation techniques as described above. The cement should be inserted in a retrograde fashion approximately 2 to 4 minutes after the start of mixing. A small catheter can be placed in the canal during this process to minimize the risk of air or fluid trapping within the cement. The cement pressurizer is applied while continuous pressure is applied for 30 to 60 seconds, which further helps with the interdigitation of the cement to the bone interface. The extruding cement should be removed. More cement can be added at this time if needed. Next, the stem is inserted slowly with close attention to the stem's version and abduction. The stem should not be rotated once it is inserted because this can distort the cement mantle. The cement should be allowed to fully harden with the implant in place before proceeding with hip reduction.

**Figure 3:**
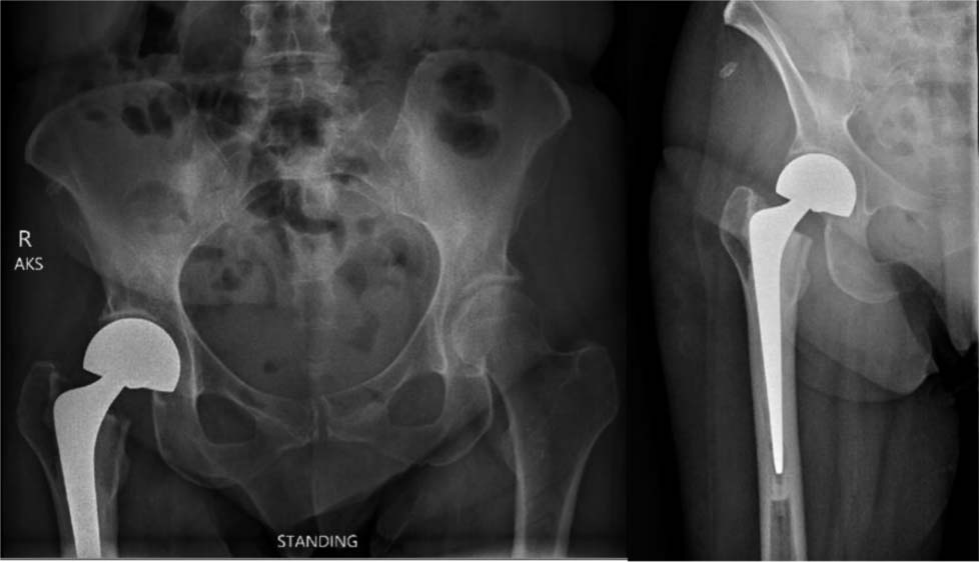
Postoperative anterior-posterior pelvis (left) and hip (right) radiograph demonstrating a cemented total hip arthroplasty with complete filling of the medullary canal.

Postoperatively, a low anterior-posterior pelvis radiograph is obtained in the postanesthesia care unit (Figure 3). Barrack et al proposed a grading system to evaluate the femoral stem cementation. A well-cemented stem is confirmed by a medullary canal that is completely filled with cement without radiolucent lines between the bone and cement.^27^ For tapered wedge-type stems, the cement should extend distal to the tip of the femoral stem with a cement mantle of 2 to 3 mm in size.

## Summary

Femoral neck fragility fractures afflict a large number of geriatric patients annually, and cemented hemiarthroplasty is the optimum treatment for these injuries. Despite the volume of supporting evidence, only half of all hemiarthroplasties implanted for femoral neck fragility fractures in the United States are cemented. There are likely several explanations, but lack of familiarity and training are strong contributors. With wider adoption by practicing orthopaedic surgeons, resident and fellow trainees will gain more opportunities to enhance their familiarity with the technique. The techniques and tips described in this article have been successful at our institution and could easily be adopted by surgeons less comfortable with the procedure.
